# Sleep duration, sleep disturbances and skeletal muscle mass change over time: A population-based longitudinal analysis in Peru

**DOI:** 10.12688/wellcomeopenres.23077.5

**Published:** 2025-03-18

**Authors:** Renzo A. Agurto-García, Enrique S. Nuñez-del-Arco, Rodrigo M. Carrillo-Larco, J. Jaime Miranda, Antonio Bernabe-Ortiz

**Affiliations:** 1 Universidad Cientifica del Sur, Lima, Peru; 2 Emory University Hubert Department of Global Health, Atlanta, Georgia, USA; 3 Emory Global Diabetes Research Center, Emory University, Atlanta, Georgia, USA; 4 CRONICAS Center of Excellence in Chronic Diseases, Universidad Peruana Cayetano Heredia, Lima, Peru

**Keywords:** Skeletal muscle, sleep duration, sleep hygiene, sleep disturbances.

## Abstract

**Background:**

The skeletal muscle has mainly a structural function and plays a role in human’s metabolism. Besides, the association between sleep quality and muscle mass, in the form of sarcopenia, has been reported. This study aimed to assess whether changes of skeletal muscle mass (SMM) over time are associated with baseline sleep duration and disturbances in a resource-constrained adult Peruvian population.

**Materials and Methods:**

Secondary analysis using information of a population-based intervention. The outcome was SMM assessed using bioimpedance and the second version of the Lee’s formula. The exposures were baseline self-reported sleep duration (normal, short and long sleepers) and disturbances (sleep difficulties and awakening at nights). Crude and adjusted linear mixed models were used to assess the associations of interest, and coefficients (β) and 95% confidence intervales (95% CI) were reported.

**Results:**

Data from 2,310 individuals at baseline, mean age 43.4 (SD: 17.2), and 1,163 (50.4%) females were analyzed. Sleep duration was 7.8 (SD: 1.3) hours/day, with 15.3% short sleepers and 11.6% long sleepers, whereas 24.2% reported sleep difficulties and 25.1% awakening at nights. In multivariable model, SMM among short and long sleepers did not vary significantly over time using the Lee’s formula; however, SMM was lower at the end of follow-up for long sleepers using bioimpedance (-0.26 kg; 95% CI: -0.47 to -0.06). Sleep disturbances were associated with a gradual SMM reduction: 0.36 kg using bioimpedance and 0.25 kg using the formula at the end of follow-up.

**Conclusions:**

Using bioimpedance and formula estimations, sleep disturbances were associated with a reduction of SMM over a period of 2.4 years. Regarding sleep duration, no SMM changes over time were seen in short sleepers, but findings were discordant in long sleepers: a reduction of SMM using bioimpedance, but no change using the formula.

## Plain Language Summary

Sleep characteristics (duration of sleep) and disturbances (sleep difficulties and awakening at nights) have been associated with the reduction of the amount of muscle mass and its strength. In this study, using two different methods to evaluate the amount of muscle mass (by formula and using bioimpedance), we found there is an inverse association between sleep disturbances and muscle mass over time among adult subjects in a resource-constrained setting in Peru. In the case of sleep duration, no muscle mass changes over time were found in short sleepers, but findings were discordant in the case of long sleepers: a reduction of muscle mass was seen using bioimpedance, but no change was seen using the formula.

## Introduction

The skeletal muscle has different functions, including produce movement, sustain body posture and position, maintain body temperature, and stabilize joints, but it also plays an important role in human metabolism as it is the main tissue responsible for insulin-mediated glucose storage, in addition to consuming a significant amount of oxygen ^1^. The loss of skeletal muscle and its strength is known as sarcopenia ^2^, whose prevalence worldwide has been estimated at 10% ^3^, and it is associated with higher rate of falls in the elderly, frailty syndrome and disability ^4^. Consequently, as the population ages, it would not take long to become a global public health concern ^5^.

Ageing has a direct effect on the loss of skeletal muscle mass (SMM) in humans ^6^; however, the reduction of skeletal muscle mass is also related to a variety of factors including malnutrition ^7^, low physical activity ^8^, and sleep patterns, including sleep duration and quality ^9, 10^. The association between sleep quality and sarcopenia has been reported as part of a systematic review of cross-sectional studies conducted using data from Germany, Taiwan, China, Japan, and Korea ^10^. This review highlighted the lack of longitudinal information assessing the association between sleep patterns and skeletal muscle mass in resource-constrained settings. Subjects typically start to lose muscle mass and strength in their 30 or 40 years, but the rate of decline increases during elderly. Thus, most of the studies have been focused on sarcopenia on elderly individuals ^10^. instead of reduction of muscle mass in the general adult population. On the other hand, a previous study involving Asian ethnic groups showed an association between sleep duration and SMM in the form of sarcopenia ^9^; however, muscle composition may be different in other resource-constrained settings.

A study in Brazil evaluating the trends of sleep complaints at the population level in Sao Paulo, reported a progressive increase of sleep complaints throughout three consecutive decades ^11^. Although in Peru, on average, the duration of sleep seems to be appropriate ^12^, there is a significant proportion of subjects with short and long sleep duration, in urban and rural areas, respectively. Peru is also a country with high obesity risk and high overweight prevalence ^13^, especially in coastal population ^14^. Sleep disturbances have been associated with obesity risk ^15, 16^, and the relationship between these two variables could increase the risk of lower SMM and an elevated risk for developing muscle diseases such as sarcopenia ^17^. Additionally, sleep and its implications for overall human health are not always considered as relevant as they should be ^18^, despite observed changes in the number of hours spent sleeping ^19^.

Consequently, this study aimed to assess the association between sleep duration and disturbances (sleep difficulties and awakening at nights) and changes of SMM over time in a resource-constrained Peruvian population.

## Methods

### Study design

The report of this manuscript followed STROBE checklist for observational studies ( *See Extended data*). Secondary data analysis using information available of a complex population-based intervention, whose details have been published elsewhere ^20^. Briefly, a stepped wedge, cluster, randomized trial was carried out to deliver a salt substitute containing sodium (75%) and potassium (25%) chloride and determine its effects on blood pressure at the population level ^21^. In the study, evaluations of the total participants were conducted at baseline and each follow-up, every 5 months starting from baseline. The study was undertaken between April 2014 (baseline) and March 2017 (last assessment). For this report, information from baseline and the five subsequent evaluations were analyzed.

### Study setting and participants

The study was conducted in Tumbes, a coastal region in northern Peru, in the border with Ecuador, with 225 thousand inhabitants, and where approximately 90% of the population is urban. Additionally, nearly 20% and 1% of the population are considered poor and extremely poor, respectively, and 15% do not have basic sanitation services ^22^.

Mid-sized villages with 350–700 individuals (~130–250 households) were initially selected for the study. Of the 20 mid-size villages available with these characteristics, six were randomly selected. After that, a census strategy was applied to invite and enroll potential participants. Eligible subjects were men and women aged ≥18 years old, full-time residents in the area, capable of understanding procedures and providing informed consent. Subjects with a self-reported history of chronic kidney disease or heart disease treated with digoxin were excluded from the study. For this analysis, individuals with complete data in the variables of interest (sleep duration and disturbances), and variables to estimate SMM were included.

### Sample size

With at least 360 participants in each sleep group (i.e., those with and without sleep difficulties), we had a power over 90% to detect a difference in SMM of 0.5 Kg (i.e., 18.3 kg among those without sleep difficulties vs. 17.7 Kg among those with sleep difficulties) at the end of follow-up, assuming a SD of 4.1 and a correlation between follow-up measurements of 0.7. Since our database had 2310 participants at baseline, we ensure enough power to find differences in our analysis.

### Definition of variables

*Outcomes:* The outcome was SMM, evaluated at baseline and each of the evaluations at follow-up, using two different approaches. The first one used an estimation of SMM based on the Rangel Peniche *et al.* formula ^23^:


SMM(Kg)=(0.05376+0.2394xResistanceindex+2.708xsex+0.65xweight)

Where: sex was represented as 0 for female and 1 for male, and the weight was measured in kilograms. The resistance index was measured in Ohms obtained by the TANITA TBF-300A Total Body Composition Analyzer, different from the one used in the formula study, but not altering findings ^24^. A standard of procedures (SOP) was created in Spanish based on the manufacturer’s manual of the TANITA TBF-300A. The result was expressed in kilograms and analyzed as numerical variable.

The second approach used the second version of the Lee’s formula, validated for Latin American population ^25^. This formula utilizes the sex, age, weight and height of participants, using the following formula:


SMM(Kg)=(0.244xweight+7.8xheight+6.6xsex+0.098xage−3.3)

Where: sex was represented as 0 for female and 1 for male, the weight was measured in kilograms (kg), height in meters, and age in years. Similarly, the result was expressed in kg and analyzed as numerical variable.

*Exposures:* Two were the independent (i.e., exposure) variables used in the analysis: sleep duration and sleep disturbances (sleep difficulties and awakening at nights). Sleep duration was evaluated utilizing the question: *“On average, in the last 6 months, how many hours a day did you sleep (including naps)?”*. This question was adapted from the Sleep Heart Health Study questionnaire ^26^, and the number of hours reported was further categorized based on the National Sleep Foundation recommendations ^27^. Therefore, in adults aged <65 years, the sleep duration was classified as normal when the subjects reported sleeping between 7 to 9 hours, as short sleepers when <7 hours, and long sleepers when >9 hours; whereas, among older adults (≥65 years), the normal sleepers slept between 7 and 8 hours, short sleepers <7 hours, and long sleepers >8 hours.

Sleep disturbances were defined using two different questions previously used in the literature ^28– 30^, and based on the Spanish version of the Obstructive Sleep Apnea questionnaire ^31^: *“During the last month, have you had difficulty falling asleep?*” and *“During the last month, how frequently have you awakened up during the night?”*. Possible responses to both questions were *almost never*, *sometimes*, and *frequently*. For the analysis, those answering *sometimes* or *frequently* were classified as having sleep disturbances, whereas those responding *almost never* were considered not having them.

*Covariables:* Demographic variables were also considered as potential confounders: sex (female vs. male), age (<30, 30–39, 40–49, 50–59, and 60+ years), education level (primary [<7 years], secondary [7–11 years], and superior [12+ years]), and socioeconomic level, using a wealth index, a composite measure of a household’s cumulative living standard, calculated using easy-to-collect data on household’s assets (TV, fridge, bicycle, etc.) and facilities, then placing them on a continuous scale of relative wealth, and finally split into tertiles (low, middle, and high) ^32^. Behavioral and anthropometric variables included in the analysis were daily smoking, defined as the self-report of the consumption of at least one cigarette per day (yes vs. no); alcohol consumption, defined as one or more episodes per month of consumption of six or more alcoholic drinks or equivalent (yes vs. no); physical activity, based on the short version of the International Physical Activity Questionnaire (IPAQ) and categorized in low vs. moderate/high levels; as well as obesity, based on body mass index ≥30 kg/m ^2^, and hypertension defined as systolic blood pressure ≥140 mmHg or diastolic blood pressure ≥90 mmHg or self-report of previous diagnosis. The presence of depressive symptoms, evaluated using the PHQ-9, a tool with nine questions and categorized into three groups (no need of treatment [0-4 points], physician judgment about treatment [5-14 points], and warrant treatment [15+ points], was also included. Finally, the original intervention (i.e., the implementation of a salt substitute) exposed villages at different moments and may have influenced our results; thus, this variable was also included in the analysis (yes vs. no).

### Procedures and data collection

A training course for fieldworkers focused on informed consent, questionnaires application, and procedures was carried out before any fieldwork activities. After providing consent, each study subject was asked to provide data about sociodemographic, lifestyle behaviors, and anthropometric measurements. This information was collected using paper-based templates. Questionnaires were created using previously validated tools such as the WHO STEPwise approach to NCD risk factor surveillance (STEPS) for lifestyles, the Sleep Heart Health Study, the Obstructive Sleep Apnea questionnaire, the International Physical Activity Questionnaire, and the Patient Health Questionnaire-9. Follow-up evaluations were carried out every five months using the same protocol and tools. Height and weight were assessed using standardized procedures. Height was measured using a stadiometer, with the participant in standing position and without shoes. The Frankfurt’s plane was used to guarantee appropriate position of the head. On the other hand, wight was obtained from the TANITA TBF-300A Total Body composition analyzer, in standing position, and without shoes. Blood pressure was performed with participants seated, after 5-min resting, using an automated device. Three measurements, at least one minute apart, were taken, and the average of the last two assessments was used for analysis.

### Statistical analysis

Analysis was conducted using STATA 16 for Windows (StataCorp, College Station, TX, US). First, a description of the study sample was carried out using mean and standard deviation (SD) for numerical variables, and absolute and relative frequencies for categorical variables. The proportion of sleep duration (i.e., short and long sleepers) as well as sleep disturbances was also estimated. In addition, the correlation between both approaches to estimate SMM was calculated using Pearson’s rho (ρ). Comparisons between demographic, behavioral and anthropometric variables according to sleep duration and disturbances were performed using Chi-squared test. On the other hand, comparisons according to SMM were assessed using t-test or analysis of variance (ANOVA) accordingly.

The associations of interest were evaluated using linear mixed models. Crude and adjusted models were built to determine baseline differences, but also changes of SMM over time. In that sense, our models included an interaction term between the exposure of interest (i.e., sleep duration, sleep difficulties, or awakening at nights) and time, assessed as categorical variable (i.e., first follow-up, second follow-up, etc.). Thus, reported coefficients (β) at baseline implied the average SMM (in Kg.) using normal sleepers estimates as the reference group, whereas, during follow-up, coefficients indicated change of SMM compared to the baseline value, for each specific sleep group. In addition, 95% confidence intervals (95%CI) were also reported. Finally, as SMM and sleep duration and disturbances differ between men and women, we evaluated if sex was an effect modifier (i.e., interaction) of the associations of interest.

## Results

### Characteristics of the study population

A total of 2,376 subjects were enrolled in the original study; however, 66 were excluded from analysis as they did not have complete information to estimate SMM [n = 62], sleep duration [n = 3], and sleep disturbances [n = 1]. Thus, data from 2,310 individuals at baseline, mean age 43.4 (SD: 17.2), 1,163 (50.4%) females and 447 (19.4%) with 12+ years of education, were analyzed.

### Sleep duration and disturbances: prevalence and associated factors

On average, reported sleep duration at baseline was 7.8 (SD: 1.3) hours per day, with 353 (15.3%; 95% CI: 13.8% - 16.8%) being classified as short sleepers and 269 (11.6%; 95% CI: 10.4% - 13.0%) as long sleepers. Table 1 shows differences in sleep duration by sex, age, education level, daily smoking, physical activity, hypertension status, and depressive symptoms.

**Table 1.** Baseline sociodemographic and lifestyle characteristics of the study population by sleep duration.

	Sleep duration			
	Normal sleepers	Short sleepers	Long sleepers	p *
	(n = 1,688)	(n = 353)	(n = 123)	
**Sex**				<0.001
Female	894 (53.0%)	123 (34.8%)	146 (54.3%)	
Male	794 (47.0%)	230 (65.2%)	123 (45.7%)	
**Age**				<0.001
<30 years	476 (28.2%)	50 (14.2%)	85 (31.6%)	
30 – 39 years	407 (24.1%)	73 (20.7%)	42 (15.6%)	
40 – 49 years	313 (18.5%)	71 (20.1%)	33 (12.3%)	
50 – 59 years	238 (14.1%)	75 (21.2%)	18 (6.7%)	
60+ years	254 (15.1%)	84 (23.8%)	91 (33.8%)	
**Education level**				<0.001
<7 years	547 (32.4%)	139 (39.4%)	127 (47.2%)	
7 – 11 years	798 (47.3%)	141 (39.9%)	111 (41.3%)	
12+ years	343 (20.3%)	73 (20.7%)	31 (11.5%)	
**Socioeconomic level**				0.63
Low	493 (29.7%)	93 (27.0%)	84 (31.7%)	
Middle	553 (33.4%)	116 (33.7%)	92 (34.7%)	
High	612 (36.9%)	135 (39.2%)	89 (33.6%)	
**Daily smoking**				0.009
No	1,597 (94.6%)	319 (90.4%)	254 (94.4%)	
Yes	91 (5.4%)	34 (9.6%)	15 (5.6%)	
**Alcohol consumption**				0.46
Low	1,588 (94.1%)	326 (92.4%)	251 (93.3%)	
High	100 (5.9%)	27 (7.7%)	18 (6.7%)	
**Physical activity**				0.004
Low	1,120 (66.4%)	216 (61.2%)	199 (74.0%)	
Moderate/high	568 (33.6%)	137 (38.8%)	70 (26.0%)	
**Obesity**				0.17
No	1,267 (75.1%)	258 (73.1%)	214 (79.6%)	
Yes	421 (24.9%)	95 (26.9%)	55 (20.4%)	
**Hypertension**				<0.001
No	1,417 (84.0%)	268 (75.9%)	205 (76.2%)	
Yes	271 (16.0%)	85 (24.1%)	64 (23.8%)	
**Depressive symptoms**				< 0.001
0–4 points	1,513 (89.7%)	283 (80.2%)	228 (84.8%)	
5–14 points	146 (8.7%)	59 (16.7%)	38 (14.1%)	
15+ points	28 (1.6%)	11 (3.1%)	3 (1.1%)	

* Comparisons were done using Chi-squared test.

Regarding sleep disturbances, 558 (24.2%; 95% CI: 22.4% - 26.0%) reported sleep difficulties during the last month, and 580 (25.1%; 95% CI: 23.4% - 26.9%) reported awakening at nights. Table 2 shows variables associated with sleep disturbances; thus, sex, age, education level, physical activity, hypertension, and depressive symptoms were strongly associated with both sleep difficulties and awakening at nights.

**Table 2.** Baseline sociodemographic and lifestyle characteristics of the study population by sleep disturbances.

	Sleep difficulties			Awakening at nights		
	No	Yes	p *	No	Yes	p *
	(n = 1,752)	(n = 558)		(n = 1,730)	(n = 580)	
**Sex**			<0.001			<0.001
Female	826 (47.2%)	337 (60.4%)		815 (47.1%)	348 (60.0%)	
Male	926 (52.9%)	221 (39.6%)		915 (52.9%)	232 (40.0%)	
**Age**			<0.001			<0.001
<30 years	522 (29.8%)	89 (15.9%)		519 (30.0%)	92 (15.9%)	
30 – 39 years	420 (24.0%)	102 (18.3%)		414 (23.9%)	108 (18.6%)	
40 – 49 years	315 (19.0%)	102 (18.3%)		312 (18.0%)	105 (18.1%)	
50 – 59 years	243 (13.9%)	88 (15.8%)		238 (13.8%)	93 (16.0%)	
60+ years	252 (14.3%)	177 (31.7%)		247 (14.3%)	182 (31.4%)	
**Education level**			<0.001			<0.001
<7 years	542 (30.9%)	271 (48.6%)		532 (30.7%)	281 (48.4%)	
7 – 11 years	854 (48.8%)	196 (35.1%)		844 (48.8%)	206 (35.5%)	
12+ years	356 (20.3%)	91 (16.3%)		354 (20.5%)	93 (16.0%)	
**Socioeconomic level**			0.26			0.38
Low	513 (29.8%)	157 (28.6%)		502 (29.6%)	168 (29.5%)	
Middle	588 (34.2%)	173 (31.6%)		582 (34.3%)	179 (31.5%)	
High	618 (36.0%)	218 (39.8%)		614 (36.2%)	222 (39.0%)	
**Daily smoking**			0.33			0.40
No	1,641 (93.7%)	529 (94.8%)		1,621 (93.7%)	549 (94.7%)	
Yes	111 (6.3%)	29 (5.2%)		109 (6.3%)	31 (5.3%)	
**Alcohol consumption**			0.05			0.01
Low	1,632 (93.1%)	533 (95.5%)		1,609 (93.0%)	556 (95.9%)	
High	120 (6.9%)	25 (4.5%)		121 (7.0%)	24 (4.1%)	
**Physical activity**			<0.001			<0.001
Low	1,118 (63.8%)	417 (74.7%)		627 (36.2%)	148 (25.5%)	
Moderate/high	634 (36.2%)	141 (25.3%)		1,103 (63.8%)	432 (74.5%)	
**Obesity**			0.57			0.40
No	1,324 (75.6%)	415 (74.4%)		1,310 (75.7%)	429 (74.0%)	
Yes	428 (24.4%)	143 (25.6%)		420 (24.3%)	151 (26.0%)	
**Hypertension**			<0.001			<0.001
No	1,501 (85.7%)	389 (69.7%)		1,484 (85.8%)	406 (70.0%)	
Yes	251 (14.3%)	169 (30.3%)		246 (14.2%)	174 (30.0%)	
**Depressive symptoms**			<0.001			<0.001
0–4 points	1,683 (96.0%)	341 (61.2%)		1,661 (96.0%)	363 (62.7%)	
5–14 points	47 (2.7%)	196 (35.2%)		48 (2.8%)	195 (33.7%)	
15+ points	22 (1.3%)	20 (3.6%)		21 (1.2%)	21 (3.6%)	

* Comparisons were done using Chi-squared test.

### Skeletal muscle mass at baseline and associated factors

At baseline, SMM was estimated at 18.2 (SD: 4.1) and 25.1 (SD: 6.0) Kg by bioimpedance and Lee’s formula, respectively (Pearson’s ρ = 0.86, p<0.001). SMM at baseline was higher among males, younger individuals, those with greater education level, and with higher socioeconomic level, using both approaches, but means were lower using bioimpedance estimates. Similarly, SMM was higher among those smoking daily, those with high alcohol consumption, those with moderate/high physical activity, and obese subjects; however, SMM was associated with hypertension using the Lee’s formula but not bioimpedance estimates. In addition, SMM was lower among those with a PHQ-9 score on 5-14 points using both approaches. Finally, in the case of sleep duration, SMM was greater in short sleepers, but lower among long sleepers, whereas when assessing sleep disturbances, both those with sleep difficulties and those awakening at nights had lower SMM ( Table 3).

**Table 3.** Baseline skeletal muscle mass (SMM, in kg) defined by bioimpedance and formula, according to sociodemographic, lifestyles and sleep duration and disturbances.

	SMM by bioimpedance (kg)		SMM by Lee’s formula (kg)	
		p-value *		p-value *
	Mean (SD)		Mean (SD)	
**Sex**		<0.001		<0.001
Female	15.1 (2.5)		20.6 (3.7)	
Male	21.3 (2.8)		29.6 (4.1)	
**Age**		<0.001		<0.001
<30 years	17.2 (4.0)		26.2 (5.7)	
30 – 39 years	18.2 (4.1)		26.4 (5.7)	
40 – 49 years	19.0 (4.1)		26.2 (5.5)	
50 – 59 years	18.9 (4.1)		24.7 (5.7)	
60+ years	18.1 (4.1)		21.1 (5.6)	
**Education level**		0.02		<0.001
<7 years	18.1 (4.0)		23.0 (6.0)	
7 – 11 years	18.4 (4.1)		26.3 (5.6)	
12+ years	17.7 (4.2)		25.9 (6.0)	
**Socioeconomic level**		0.03		<0.001
Low	18.0 (3.9)		24.3 (6.0)	
Middle	18.1 (4.1)		25.1 (5.9)	
High	18.5 (4.3)		25.8 (6.0)	
**Daily smoking**		<0.001		<0.001
No	18.0 (4.1)		24.8 (6.0)	
Yes	21.0 (2.6)		29.1 (4.1)	
**Alcohol consumption**		<0.001		<0.001
Low	17.9 (4.1)		24.7 (5.9)	
High	21.3 (3.0)		30.8 (4.0)	
**Physical activity**		<0.001		<0.001
Low	17.5 (4.0)		24.0 (5.9)	
Moderate/high	19.5 (4.0)		27.2 (5.5)	
**Obesity**		<0.001		<0.001
No	17.6 (3.9)		24.3 (5.8)	
Yes	19.8 (4.1)		27.4 (6.1)	
**Hypertension**		0.18		<0.001
No	18.1 (4.1)		25.5 (5.8)	
Yes	18.4 (4.4)		23.1 (6.6)	
**Depressive symptoms**		<0.001		<0.001
0–4 points	18.3 (4.1)		25.4 (5.9)	
5–14 points	17.2 (4.1)		22.4 (6.4)	
15+ points	18.3 (3.8)		26.0 (5.7)	
**Sleep duration**		<0.001		<0.001
Normal sleepers	17.9 (4.0)		25.0 (5.8)	
Short sleepers	19.7 (4.5)		26.4 (6.4)	
Long sleepers	17.5 (3.9)		23.7 (6.2)	
**Sleep difficulties**		0.003		<0.001
No	18.3 (4.1)		25.6 (5.9)	
Yes	17.7 (4.2)		23.3 (5.9)	
**Awakening at nights**		0.004		<0.001
No	18.3 (4.1)		25.7 (5.9)	
Yes	17.7 (4.2)		23.4 (5.9)	

* P-values were estimated using T test or analysis of variance, accordingly.

### Variation of skeletal muscle mass over time

During the 2.4 (SD: 0.1) years of follow-up, rate of attrition was 12.1% (from 2,310 at baseline to 2031 at the end of follow-up). Those who were lost to follow-up were younger, had higher education, had low levels of physical activity, had BMI < 30 kg/m ^2^, and had more depressive symptoms at baseline than those who were kept in the study.

Regarding sleep duration, according to multivariable model, and compared to normal sleepers, short sleepers had higher SMM at baseline using both SMM approaches, whereas SMM of long sleepers were similar to normal sleepers ( Table 4). Over time and compared to SMM at baseline, normal sleepers showed high SMM variability using both approaches, but changes were not significant at the end of follow-up. SMM in short sleepers did not vary significantly over time; however, SMM was lower among long sleepers at the end of follow-up using bioimpedance (-0.26; 95% CI: -0.47 to -0.06), but not using the Lee’s formula. Sex was not an effect modifier of the association when using any of the approaches for SMM estimations.

**Table 4.** Variation of SMM, defined by bioimpedance and formula, over time according to sleep duration: adjusted models.

	SMM by bioimpedance	SMM by formula
	Adjusted model *	Adjusted model *
	β (95% CI)	β (95% CI)
**Sleep duration at baseline**		
Normal sleepers	Reference	Reference
Short sleepers	**0.42 (0.09 to 0.76)**	**0.34 (0.15 to 0.52)**
Long sleepers	-0.12 (-0.49 to 0.25)	-0.16 (-0.64 to 0.32)
**Normal sleepers during follow-up**		
First follow-up	**0.18 (0.04 to 0.33)**	**0.15 (0.11 to 0.19)**
Second follow-up	0.01 (-0.14 to 0.15)	**-0.10 (-0.17 to -0.04)**
Third follow-up	0.16 (-0.05 to 0.36)	-0.06 (-0.16 to 0.04)
Fourth follow-up	0.23 (-0.02 to 0.48)	**-0.08 (-0.14 to -0.01)**
Fifth follow-up	0.23 (-0.06 to 0.51)	0.03 (-0.06 to 0.11)
**Short sleepers during follow-up**		
First follow-up	-0.04 (-0.27 to 0.18)	-0.01 (-0.16 to 0.13)
Second follow-up	-0.07 (-0.44 to 0.31)	0.01 (-0.10 to 0.12)
Third follow-up	-0.07 (-0.36 to 0.22)	-0.04 (-0.16 to 0.08)
Fourth follow-up	-0.12 (-0.43 to 0.20)	-0.06 (-0.13 to 0.01)
Fifth follow-up	-0.18 (-0.47 to 0.11)	-0.08 (-0.17 to 0.01)
**Long sleepers during follow-up**		
First follow-up	0.03 (-0.04 to 0.09)	-0.01 (-0.09 to 0.06)
Second follow-up	-0.08 (-0.24 to 0.08)	-0.04 (-0.13 to 0.04)
Third follow-up	-0.03 (-0.21 to 0.15)	-0.09 (-0.21 to 0.04)
Fourth follow-up	-0.05 (-0.29 to 0.19)	-0.11 (-0.24 to 0.03)
Fifth follow-up	**-0.26 (-0.47 to -0.06)**	-0.03 (-0.22 to 0.17)

Coefficients are interpreted as change of SMM (in kg). * Model adjusted for sex, age, education level, socioeconomic status, daily smoking, alcohol consumption, physical activity, obesity, hypertension status, depressive symptoms, and intervention during follow-up.

Regarding sleep disturbances, in multivariable model ( Table 5), results depended on SMM approach used. SMM was higher at baseline among those having sleep difficulties, but there was no difference using the Lee’s formula. Over time, those with sleep difficulties had a gradual SMM reduction using both approaches, but such reduction was greater using bioimpedance (0.36 kg; 95% CI: 0.26 to 0.45) than the formula (0.24 kg; 95% CI: 0.12 to 0.35) at the end of follow-up. Sex was an effect modifier of the association between sleep disturbances and SMM change over time. Thus, the drop of SMM at the end of follow-up was greater among men when using bioimpedance (0.50 kg; 95% CI: 0.34 to 0.68) than women (0.20 kg; 95% CI: 0.01 to 0.39). However, in the case of using the Lee’s formula, the drop of SMM at the end of follow-up was greater among women (0.28 kg; 95% CI: 0.16 to 0.39) than among men (0.15 kg; 95% CI: -0.32 to 0.01).

**Table 5.** Variation of skeletal muscle mass over time according to sleep disturbances: adjusted.

	SMM by bioimpedance	SMM by formula
	Adjusted model *	Adjusted model *
	β (95% CI)	β (95% CI)
**Sleep difficulties at baseline**		
No	Reference	Reference
Yes	0.20 (-0.02 to 0.42)	-0.04 (-0.38 to 0.31)
**Without sleep difficulties during follow-up**		
First follow-up	**0.21 (0.07 to 0.35)**	**0.16 (0.14 to 0.19)**
Second follow-up	0.04 (-0.09 to 0.17)	**-0.08 (-0.13 to -0.02)**
Third follow-up	**0.18 (0.02 to 0.35)**	-0.03 (-0.12 to 0.06)
Fourth follow-up	**0.27 (0.03 to 0.51)**	-0.05 (-0.13 to 0.02)
Fifth follow-up	0.26 (-0.02 to 0.53)	0.07 (-0.03 to 0.17)
**With sleep difficulties during follow-up**		
First follow-up	**-0.14 (-0.25 to -0.03)**	-0.05 (-0.10 to 0.01)
Second follow-up	**-0.23 (-0.42 to -0.04)**	**-0.13 (-0.18 to -0.07)**
Third follow-up	**-0.15 (-0.25 to -0.05)**	**-0.18 (-0.27 to -0.10)**
Fourth follow-up	-0.26 (-0.55 to 0.04)	**-0.18 (-0.29 to 0.08)**
Fifth follow-up	**-0.36 (-0.45 to -0.26)**	**-0.24 (-0.35 to -0.12)**
	Adjusted model *	Adjusted model *
	β (95% CI)	β (95% CI)
**Awakenings at night at baseline**		
No	Reference	Reference
Yes	0.17 (-0.05 to 0.40)	-0.04 (-0.38 to 0.30)
**Without awakening at night during follow-up**		
First follow-up	**0.20 (0.06 to 0.35)**	**0.16 (0.14 to 0.18)**
Second follow-up	0.04 (-0.10 to 0.18)	**-0.08 (-0.13 to -0.02)**
Third follow-up	**0.19 (0.03 to 0.35)**	-0.02 (-0.11 to 0.06)
Fourth follow-up	**0.28 (0.05 to 0.51)**	-0.05 (-0.12 to 0.02)
Fifth follow-up	0.26 (-0.02 to 0.54)	0.07 (-0.02 to 0.17)
**With awakening at night during follow-up**		
First follow-up	-0.09 (-0.25 to 0.06)	-0.04 (-0.09 to 0.01)
Second follow-up	-0.22 (-0.44 to 0.01)	**-0.13 (-0.17 to -0.08)**
Third follow-up	**-0.16 (-0.26 to -0.06)**	**-0.19 (-0.28 to -0.10)**
Fourth follow-up	**-0.27 (-0.53 to -0.01)**	**-0.20 (-0.29 to -0.10)**
Fifth follow-up	**-0.36 (-0.45 to -0.27)**	**-0.25 (-0.35 to -0.15)**

Coefficients are interpreted as change of SMM (in kg). * Model adjusted for sex, age, education level, socioeconomic status, daily smoking, alcohol consumption, physical activity, obesity, hypertension status, depressive symptoms, and intervention during follow-up.

Regarding awakening at nights, SMM was similar at baseline using both approaches ( Table 5), with a steady reduction of SMM over time with a greater drop with bioimpedance (0.36 kg; 95% CI: 0.27 to 0.45) compared to the Lee’s formula (0.25 kg; 95% CI: 0.15 to 0.35) at the end of follow-up. Sex was an effect modifier of the association between awakenings at nights and SMM change over time. Thus, the drop of SMM at the end of follow-up was greater among men (0.52 kg; 95% CI: 0.33 to 0.70) than women (0.19 kg; 95% CI: 0.02 to 0.36) when using bioimpedance. However, in the case of using the Lee’s formula, the drop of SMM at the end of follow-up was slightly greater among women (0.27 kg; 95% CI: 0.17 to 0.36) than among men (0.20 kg; 95% CI: 0.03 to 0.37).

## Discussion

### Main findings

This study found that there is an inverse association between sleep disturbances (i.e., sleep difficulties and awakening at nights) and SMM over time among adult individuals in a resource-constrained setting in Peru. Moreover, two different approaches to estimate SMM showed similar results, although overestimated in the case of bioimpedance. In the case of sleep duration, results showed no SMM changes over time in short sleepers, but findings were discordant in the case of long sleepers: a reduction of SMM was seen using bioimpedance, but no SMM change was observable using the Lee’s formula. Moreover, sex was not an effect modifier in the case of sleep duration and SMM variation over time, but it was for sleep difficulties and awakening at nights. In these latter cases, reduction of SMM was greater among men when using bioimpedance, but such drop was greater among women when using Lee’s formula.

### Comparison with previous studies

A previous systematic review of six cross-sectional studies has reported that having inadequate sleep, assessed using sleep quality, was associated with high prevalence of sarcopenia compared to those with adequate sleep ^10^. These studies were diverse in methodology as muscle mass was measured using dual-energy X-ray absorptiometry (DEXA) in three studies, bioimpedance in two studies, and self-reporting in only one study. DEXA and bioimpedance may be difficult to utilize in resource-constrained settings, and DEXA might overestimate sarcopenia ^33^. Moreover, only four studies provided sex-dependent data and not significant differences were observed, perhaps due to the high heterogeneity of the studies included ^10^.

From a longitudinal perspective, a cohort study, including data of 902 community-dwelling elderly Chinese individuals and three years follow-up, reported short sleep duration was associated with muscle mass decline ^34^. In a different Chinese cohort of 920 subjects, a U-shaped association in sarcopenia prevalence was demonstrated, with short and long sleep duration obtaining greater prevalence estimates than those with normal sleep duration ^9^. Unlike these previous studies, our results are based on bioimpedance estimates in addition to a validated formula for Latin American populations to show comparable results.

Regarding cross-sectional studies, in a sample of 488 Taiwanese elderly adults, a report showed a significant U-shaped association between sleep duration and the prevalence of sarcopenia ^35^. Thus, compared to adults with 6–8 h of sleep, elderly subjects with <6 h of sleep had a nearly 3-fold increased probability of sarcopenia, whereas elderly with ≥8 h of sleep had a nearly 2-fold increased risk of sarcopenia. Similarly, in a study conducted in Germany with 1196 elderly subjects, an association of sleep length, quality and efficiency, and low muscle mass was found, especially in men ^36^. In addition, a previous cross-sectional study has reported an association between sleep duration and skeletal muscle mass, assessed as a categorical variable in the form of sarcopenia, especially among long sleepers ^37^. Finally, a study in 915 middle-aged subjects reported that decreased sleep duration, quality, and timing were associated with osteopenia and sarcopenia ^38^. Thus, our results are in line with these studies, showing an effect of sleep disturbances on SMM in a Latin American population.

Finally, although the correlation between results by bioimpedance and the Lee’s formula was high, estimations of SMM and its changes over time were divergent in some cases. Some potential reasons for this are that the algorithm used to estimate SMM when applying bioimpedance may not be applicable to Latin America populations, and for instance increase the risk of measurement bias. That is not the case when using the Lee’s formula, previously validated for Latin American population ^25^. Besides, the bioimpedance device (TANITA TBF-300A) used for this study was not the same as the original one ^23^, implying measurement bias as different devices may use different estimation algorithms.

### Public health relevance

Our results show that sleep disturbances (i.e., sleep difficulties and awakening at nights) were associated with a loss of muscle mass: approximately 250 and 350 grams of muscle mass depending on the approach used during 2.4 years of follow-up. These results are relevant as these estimates were obtained from adult individuals (i.e., 18+ years), and not only from elderly subjects, in which these estimates may be greater. Sleep duration seems to have a potential effect on SMM, but results were not consistent according to the two approaches used. In addition to that, using bioimpedance or a formula may result in different estimates. Thus, although reduction of SMM is consistent using both approaches, the changes according to sex are contrasting.

Therefore, our findings suggest the need to maintain appropriate sleep patterns to preserve and avoid loss of muscle mass. Appropriate prevention strategies are also mandatory as usually sleep quality and duration tend to reduce during ageing, whereas time awake increased after sleep onset ^39^. For example, a trial conducted in Sao Paulo and using resistance exercise training for 12 weeks improved simultaneously sleep parameters and muscle performance ^40^. Thus, an appropriate strength-training routine could be used, involving a well-qualified trainer to help set up a detailed sequence and supervise workouts. Diet improvement is also needed, for example, adequate protein and/or amino-acid intake plus exercise can enhance the benefits. Vitamin D, omega-3s, and dairy may positively impact muscle mass and strength. On the other hand, although sleep duration seems to be similar to that reported at the national level ^12^, there is a clear need of increasing awareness about the potential effect of sleep patterns on health outcomes.

### Strengths and limitations

Our study benefits from the longitudinal analysis of data of a population-based survey, being one of the few studies conducted outside the Asian continent as reported in previous reports ^9, 34, 37^. In addition, skeletal muscle mass was estimated using two different approaches, by formula and bioimpedance analysis, in a wide age group (≥18 years) and not only among elderly individuals. Nevertheless, this study has limitations. First, the data used in the present manuscript comes from a population-based intervention using a salt substitute, which may have influenced SMM. However, we adjusted our models for the intervention and the moment at which the salt substitute was implemented as the original study had a stepped wedge design. A previous study reported that salt substitutes were believed to improve sleeping disorders and musculoskeletal health ^41^ but was considered an exaggeration of the benefits of the substitute. A different study reported that short-duration sleepers consumed insufficient amounts of vegetables but preferred salty food ^42^. Literature suggests that mechanisms playing a role in the association between sleep duration and dietary intake are likely to be multifactorial and include differences in the appetite-related hormones ^43^. Second, selection bias may be a concern as this study was conducted in a semiurban population in the north of Peru, which may not be representative of all the country or other populations. In addition, attrition rates may have an impact on our results, especially because those lost to follow-up were different from those who were kept in the study. Third, a risk of measurement bias may arise as the algorithm used by bioimpedance may not be appropriate for our population. Additionally, the bioimpedance device used for this study was not the same used in the original study ^23^, implying measurement bias as different devices may use different estimation algorithms. Fourth, sleep duration and disturbances were measured using a self-reported technique, a method that can be less accurate than actigraphy, and for instance, recall and measurement biases may arise ^44^. Finally, some confounders of the association of interest may have not been included as they were not available, such as the presence of anxiety symptoms, diet patterns, especially meat consumption, protein consumption, physical activity intensity, among others. Additionally, other important variables affecting women muscle mass were not evaluated (i.e., menopause, breastfeeding and hysterectomy surgeries) as were not available in the original study.

## Conclusion

Using bioimpedance and formula estimations, sleep disturbances (i.e., sleep difficulties and awakening at nights) were associated with a reduction of SMM over a period of 2.4 years. Regarding sleep duration, no SMM changes over time were seen in short sleepers, but findings were discordant in long sleepers: a reduction of SMM using bioimpedance, but no change using the formula. Our results suggest that maintaining adequate sleep patterns seems to be relevant for preserving muscle mass.

## Ethics and consent

The present study adhered to the Declaration of Helsinki. The protocol and informed consents were reviewed and approved by the Institutional Review Boards of the Universidad Peruana Cayetano Heredia, in Lima, Peru (SIDISI code 58563, date of approval: August 18, 2014) and the Johns Hopkins University, Baltimore, MD, US (code 00004928, date of approval: September 17, 2014). A written informed consent was read and obtained before recruitment and start of research activities. The present analysis was reviewed and approved by the Institutional Ethical Committee of Universidad Científica del Sur, Lima, Peru (code: 463-2020-PRE15, date of approval: September 02, 2022).

## Data Availability

Figshare: SALT Study - Data for skeletal muscle mass analysis, https://doi.org/10.6084/m9.figshare.26894230.v1 ^45^ This project contains the following underlying data: - SALT Study – Dataset.csv (dataset) - SALT Study - Dictionary.txt (key to variable abbreviations) Data are available under the terms of the Creative Commons Attribution 4.0 International license (CC-BY 4.0). Figshare: SALT Study – Questionnaires, https://doi.org/10.6084/m9.figshare.26950948.v1 ^46^ This project contains the following extended data: -Salt substitute - Baseline questionnaire (English).pdf (Questionnaire) -Salt substitute - Follow-up questionnaire (English).pdf (Questionnaire) Data are available under the terms of the Creative Commons Attribution 4.0 International license (CC-BY 4.0). Figshare: Sleep pattern and muscle mass – STROBE checklist, https://doi.org/10.6084/m9.figshare.27004747 ^47^ This project contains the following extended data: - Sleep pattern and muscle mass – STROBE checklist for observational studies. Data are available under the terms of the Creative Commons Attribution 4.0 International license (CC-BY 4.0).

## References

[1] Stump CS Henriksen EJ Wei Y : The metabolic syndrome: role of skeletal muscle metabolism. *Ann Med.* 2006;38(6):389–402. 10.1080/07853890600888413 17008303

[2] Cruz-Jentoft AJ Bahat G Bauer J : Sarcopenia: revised European consensus on definition and diagnosis. *Age Ageing.* 2019;48(1):16–31. 10.1093/ageing/afy169 30312372 PMC6322506

[3] Shafiee G Keshtkar A Soltani A : Prevalence of sarcopenia in the world: a systematic review and meta- analysis of general population studies. *J Diabetes Metab Disord.* 2017;16: 21. 10.1186/s40200-017-0302-x 28523252 PMC5434551

[4] Papadopoulou SK : Sarcopenia: a contemporary health problem among older adult populations. *Nutrients.* 2020;12(5): 1293. 10.3390/nu12051293 32370051 PMC7282252

[5] Beaudart C Rizzoli R Bruyère O : Sarcopenia: burden and challenges for public health. *Arch Public Health.* 2014;72(1): 45. 10.1186/2049-3258-72-45 25810912 PMC4373245

[6] Larsson L Degens H Li M : Sarcopenia: aging-related loss of muscle mass and function. *Physiol Rev.* 2019;99(1):427–511. 10.1152/physrev.00061.2017 30427277 PMC6442923

[7] Beaudart C Sanchez-Rodriguez D Locquet M : Malnutrition as a strong predictor of the onset of sarcopenia. *Nutrients.* 2019;11(12):2883. 10.3390/nu11122883 31783482 PMC6950107

[8] Meier NF Lee DC : Physical activity and sarcopenia in older adults. *Aging Clin Exp Res.* 2020;32(9):1675–87. 10.1007/s40520-019-01371-8 31625078

[9] Hu X Jiang J Wang H : Association between sleep duration and sarcopenia among community-dwelling older adults: a cross-sectional study. *Medicine (Baltimore).* 2017;96(10): e6268. 10.1097/MD.0000000000006268 28272238 PMC5348186

[10] Rubio-Arias JÁ Rodríguez-Fernández R Andreu L : Effect of sleep quality on the prevalence of sarcopenia in older adults: a systematic review with meta-analysis. *J Clin Med.* 2019;8(12):2156. 10.3390/jcm8122156 31817603 PMC6947616

[11] Santos-Silva R Bittencourt LRA Pires MLN : Increasing trends of sleep complaints in the city of Sao Paulo, Brazil. *Sleep Med.* 2010;11(6):520–4. 10.1016/j.sleep.2009.12.011 20494615

[12] Carrillo-Larco RM Bernabé-Ortiz A Miranda JJ : Peruvians' sleep duration: analysis of a population-based survey on adolescents and adults. *PeerJ.* 2014;2: e345. 10.7717/peerj.345 24765579 PMC3994633

[13] NCD Risk Factor Collaboration (NCD-RisC): Worldwide trends in Body-Mass Index, underweight, overweight, and obesity from 1975 to 2016: a pooled analysis of 2416 population-based measurement studies in 128·9 million children, adolescents, and adults. *Lancet.* 2017;390(10113):2627–42. 10.1016/S0140-6736(17)32129-3 29029897 PMC5735219

[14] Farro-Maldonado MY Gutiérrez-Pérez G Hernández-Vásquez A : Socioeconomic inequalities in Abdominal Obesity among Peruvian adults. *PLoS One.* 2021;16(7): e0254365. 10.1371/journal.pone.0254365 34288938 PMC8294571

[15] Bacaro V Ballesio A Cerolini S : Sleep duration and obesity in adulthood: an updated systematic review and meta-analysis. *Obes Res Clin Pract.* 2020;14(4):301–9. 10.1016/j.orcp.2020.03.004 32527625

[16] Tan X Chapman CD Cedernaes J : Association between long sleep duration and increased risk of obesity and type 2 diabetes: a review of possible mechanisms. *Sleep Med Rev.* 2018;40:127–34. 10.1016/j.smrv.2017.11.001 29233612

[17] Stenholm S Harris TB Rantanen T : Sarcopenic obesity: definition, cause and consequences. *Curr Opin Clin Nutr Metab Care.* 2008;11(6):693–700. 10.1097/MCO.0b013e328312c37d 18827572 PMC2633408

[18] Chattu VK Manzar MD Kumary S : The global problem of insufficient sleep and its serious public health implications. *Healthcare (Basel).* 2018;7(1):1. 10.3390/healthcare7010001 30577441 PMC6473877

[19] Hoyos C Glozier N Marshall NS : Recent evidence on worldwide trends on sleep duration. *Curr Sleep Medicine Rep.* 2015;1:195–204. 10.1007/s40675-015-0024-x

[20] Bernabe-Ortiz A Diez-Canseco F Gilman RH : Launching a salt substitute to reduce blood pressure at the population level: a cluster randomized stepped wedge trial in Peru. *Trials.* 2014;15: 93. 10.1186/1745-6215-15-93 24667035 PMC3976551

[21] Bernabe-Ortiz A Rosas VGSY Ponce-Lucero V : Effect of salt substitution on community-wide blood pressure and hypertension incidence. *Nat Med.* 2020;26(3):374–8. 10.1038/s41591-020-0754-2 32066973 PMC7613083

[22] Censos Nacionales 2017: XII de Población, VII de Vivienda y III de comunidades Indígenas. Reference Source

[23] Rangel Peniche DB Raya Giorguli G Alemán-Mateo H : Accuracy of a predictive bioelectrical impedance analysis equation for estimating appendicular Skeletal Muscle Mass in a non-Caucasian sample of older people. *Arch Gerontol Geriatr.* 2015;61(1):39–43. 10.1016/j.archger.2015.03.007 25857600

[24] Yu SCY Powell A Khow KSF : The performance of five bioelectrical impedance analysis prediction equations against dual X-ray absorptiometry in estimating appendicular Skeletal Muscle Mass in an adult Australian population. *Nutrients.* 2016;8(4):189. 10.3390/nu8040189 27043617 PMC4848658

[25] Fernandez Veitez JA Aguilera RR : [Muscle mass estimation by different anthropometric equations in high performance weightlifters]. *Archivos de Medicina del Deporte.* 2001;XVIII(86):585–591. Reference Source

[26] Zhang GQ Cui L Mueller R : The national sleep research resource: towards a sleep data commons. *J Am Med Inform Assoc.* 2018;25(10):1351–1358. 10.1093/jamia/ocy064 29860441 PMC6188513

[27] Hirshkowitz M Whiton K Albert SM : National Sleep Foundation's sleep time duration recommendations: methodology and results summary. *Sleep Health.* 2015;1(1):40–43. 10.1016/j.sleh.2014.12.010 29073412

[28] Bansil P Kuklina EV Merritt RK : Associations between sleep disorders, sleep duration, quality of sleep, and hypertension: results from the national health and nutrition examination survey, 2005 to 2008. *J Clin Hypertens (Greenwich).* 2011;13(10):739–43. 10.1111/j.1751-7176.2011.00500.x 21974761 PMC8108844

[29] Vozoris NT : The relationship between insomnia symptoms and hypertension using United States population-level data. *J Hypertens.* 2013;31(4):663–71. 10.1097/HJH.0b013e32835ed5d0 23391985

[30] Vozoris NT : Insomnia symptom frequency and hypertension risk: a population-based study. *J Clin Psychiatry.* 2014;75(6):616–23. 10.4088/JCP.13m08818 25004185

[31] Chiner E Landete P Sancho-Chust JN : Adaptation and validation of the Spanish version of OSA-18, a quality of life questionnaire for evaluation of children with Sleep Apnea-Hypopnea Syndrome. *Arch Bronconeumol.* 2016;52(11):553–559. 10.1016/j.arbres.2016.04.003 27199262

[32] United States Agency for International Development: Wealth index. MD, US: USAID,2016; [updated 2016; cited 2023 August 8]. Reference Source

[33] Quiñonez-Olivos CG Salinas-Martínez R Ortiz-Jiménez XA : Muscle mass measured using bioelectrical impedance analysis, calf circumference and grip strength in older adults. *Med Univ.* 2016;18(72):158–162. 10.1016/j.rmu.2016.06.005

[34] Fu L Yu X Zhang W : The relationship between sleep duration, falls, and muscle mass: a cohort study in an elderly Chinese population. *Rejuvenation Res.* 2019;22(5):390–398. 10.1089/rej.2018.2102 30565504

[35] Chien MY Wang LY Chen HC : The relationship of sleep duration with obesity and sarcopenia in community-dwelling older adults. *Gerontology.* 2015;61(5):399–406. 10.1159/000371847 25721246

[36] Buchmann N Spira D Norman K : Sleep, muscle mass and muscle function in older people. *Dtsch Arztebl Int.* 2016;113(15):253–60. 10.3238/arztebl.2016.0253 27151463 PMC4860870

[37] Kwon YJ Jang SY Park EC : Long sleep duration is associated with sarcopenia in Korean adults based on data from the 2008–2011 KNHANES. *J Clin Sleep Med.* 2017;13(9):1097–1104. 10.5664/jcsm.6732 28760192 PMC5566466

[38] Lucassen EA de Mutsert R le Cessie S : Poor sleep quality and later sleep timing are risk factors for osteopenia and sarcopenia in middle-aged men and women: the NEO study. *PLoS One.* 2017;12(5): e0176685. 10.1371/journal.pone.0176685 28459884 PMC5411054

[39] Pace-Schott EF Spencer RMC : Age-related changes in the cognitive function of sleep. *Prog Brain Res.* 2011;191:75–89. 10.1016/B978-0-444-53752-2.00012-6 21741545

[40] de Sá Souza H de Melo CM Piovezan RD : Resistance training improves sleep and anti-inflammatory parameters in sarcopenic older adults: a randomized controlled trial. *Int J Environ Res Public Health.* 2022;19(23): 16322. 10.3390/ijerph192316322 36498393 PMC9736460

[41] Liu Y Chu H Peng K : Factors associated with the use of a salt substitute in rural China. *JAMA Netw Open.* 2021;4(12): e2137745. 10.1001/jamanetworkopen.2021.37745 34878549 PMC8655604

[42] Shi Z McEvoy M Luu J : Dietary fat and sleep duration in Chinese men and women. *Int J Obes (Lond).* 2008;32(12):1835–40. 10.1038/ijo.2008.191 18982012

[43] Dashti HS Scheer FA Jacques PF : Short sleep duration and dietary intake: epidemiologic evidence, mechanisms, and health implications. *Adv Nutr.* 2015;6(6):648–59. 10.3945/an.115.008623 26567190 PMC4642416

[44] Cespedes EM Hu FB Redline S : Comparison of self-reported sleep duration with actigraphy: results from the Hispanic Community Health Study/Study of Latinos Sueño ancillary study. *Am J Epidemiol.* 2016;183(6):561–73. 10.1093/aje/kwv251 26940117 PMC4782764

[45] Bernabé-Ortiz A : SALT study - data for skeletal muscle mass analysis. *Figshare.* [Dataset].2024. 10.6084/m9.figshare.26894230.v1

[46] Bernabe-Ortiz A : SALT study – questionnaires. *figshare.* 2024. 10.6084/m9.figshare.26950948.v1

[47] Bernabé-Ortiz A : Sleep pattern and muscle mass - STROBE checklist. *fighshare.* 2024. 10.6084/m9.figshare.27004747

