# Supplementary material for: Sleep duration, sleep disturbances and skeletal muscle mass change over time: A population-based longitudinal analysis in Peru
Source: Wellcome Open Res. 2025 Mar 18;9:565. Originally published 2024 Oct 2. [Version 5] doi: 10.12688/wellcomeopenres.23077.5 (PMC11757916; doi:10.12688/wellcomeopenres.23077.5)
Supplement: Peer review history

# Reviewer report: Reviewer response for version 5

**DOI:** 10.21956/wellcomeopenres.26430.r120814

**Published:** 2025-03-26

**Authors:** Nurel Ertürk

**Affiliations:** 1 University of Health Sciences, Trabzon, Turkey

The changes and additions and comments were examined. However, this does not show that the results provided are reliable and reflect reality. It is not possible to talk about clear results from a study in which these confounding factors are not examined and sleep is subjectively examined. I think the method applied is not sufficient.

Is the work clearly and accurately presented and does it cite the current literature?

No

If applicable, is the statistical analysis and its interpretation appropriate?

Partly

Are all the source data underlying the results available to ensure full reproducibility?

No

Is the study design appropriate and is the work technically sound?

No

Are the conclusions drawn adequately supported by the results?

No

Are sufficient details of methods and analysis provided to allow replication by others?

Yes

Reviewer Expertise:

Sleep health, obstructive sleep apnea, physical activity

I confirm that I have read this submission and believe that I have an appropriate level of expertise to state that I do not consider it to be of an acceptable scientific standard, for reasons outlined above.

# Reviewer report: Reviewer response for version 4

**DOI:** 10.21956/wellcomeopenres.26123.r117809

**Published:** 2025-03-02

**Authors:** Nurel Ertürk

**Affiliations:** 1 University of Health Sciences, Trabzon, Turkey

The authors have not yet responded or made any edits to my comments in the text, so my initial comments continue as they are, and therefore I do not yet have a contribution to make to this study. My initial opinion continues.

Is the work clearly and accurately presented and does it cite the current literature?

No

If applicable, is the statistical analysis and its interpretation appropriate?

Partly

Are all the source data underlying the results available to ensure full reproducibility?

No

Is the study design appropriate and is the work technically sound?

No

Are the conclusions drawn adequately supported by the results?

No

Are sufficient details of methods and analysis provided to allow replication by others?

Yes

Reviewer Expertise:

Sleeo health, obstructive sleep apnea, physical activity

I confirm that I have read this submission and believe that I have an appropriate level of expertise to state that I do not consider it to be of an acceptable scientific standard, for reasons outlined above.

# Response

**Published:** 2025-03-10

**Authors:** Antonio Bernabe-Ortiz

**Affiliations:** Universidad Cientifica del Sur, Peru

We would like to apologize to the reviewer as we did not notice this new review. We have tried to respond to comments one by one. The literature and physiological background of the study are lacking. We can see this in many ways:  

***Comment:* Many factors such as hormones that will affect women's muscle mass, menopause, breastfeeding, and hysterectomy surgeries affect muscle mass. These are confounding factors when researching muscle mass. These have not been looked at.**

***Response:*** Unfortunately, as we explained in the methods section of the manuscript, this is a secondary analysis using data of a population-based intervention implementing a salt substitute in a region in Peru. Therefore, it is not usual to evaluate menopause, breastfeeding, or hysterectomy surgeries as part of an intervention like the original one.  However, we have included sex as an effect modifier (see results section). In addition, we have added sentences regarding this point in the limitations section: “ *Additionally, other important variables affecting women muscle mass were not evaluated (i.e., menopause, breastfeeding and hysterectomy surgeries) as were not available in the original study*”.  

***Comment:* In both the Summary and Results sections, only the number of female patients is written, and the number of male patients should be added.**

***Response:*** We disagree with the reviewer regarding this point. The number of male participants can be easily calculated by resting 2310 – 1163 = 1147, and the percentage is 49.6% (a simple difference of 100% - 50.4%). Moreover, the number of male participants can be calculated by adding the number in the row “male” in Table 1. As a result, we do not see mandatory adding redundant information, especially when space is limited.  

***Comment:* In the measurements made, sleep disturbance and sleep duration were evaluated completely subjectively and according to patient responses. Sleep duration has many sub parameters. Such as the hours spent in bed, the hours spent in bed and getting out of bed, whether the person walked around the house at night and stayed awake during the sleep time... It is an unreliable measurement to evaluate sleep only with subjective measurement and even with two questions. The biggest shortcoming of this study is that sleep measurements are very inadequate and unreliable.**

***Response:*** As explained before, this study was conducted in a different research context in resource-constrained settings with limited funding. We have tried to use questions previously validated or used in previous studies. For example, the question used to assess sleep duration was taken from the Sleep Heart Health Study questionnaire. In the same line, questions to evaluate sleep disturbances were taken from the Spanish version of the Obstructive Sleep Apnea questionnaire. This information has been added to the Methods section.  In addition, a sentence in the limitations section has been added regarding this point: “ *Fourth, sleep duration and disturbances were measured using a self-reported technique, a method that can be less accurate than actigraphy, and for instance, recall and measurement biases may arise*”.  

***Comment:* "Thus, our results expand literature by showing a longitudinal effect of sleep disturbances on SMM in a Latin American population." You say that there is no relationship in your results, and you also did not examine many confounding factors. Based on what conclusion do you say this?**

***Response:*** Please read carefully the results section. Regarding sleep difficulties, we said: “ *Over time, those with sleep difficulties had a gradual SMM reduction using both approaches, but such reduction was greater using bioimpedance (0.36 kg; 95% CI: 0.26 to 0.45) than the formula (0.24 kg; 95% CI: 0.12 to 0.35) at the end of follow-up*”. Regarding awakening at nights, we said: “SMM was similar at baseline using both approaches, with a steady reduction of SMM over time with a greater drop with bioimpedance (0.36 kg; 95% CI: 0.27 to 0.45) compared to the Lee’s formula (0.25 kg; 95% CI: 0.15 to 0.35) at the end of follow-up". Thus, our statement is correct; nevertheless, we have rewritten this sentence for clarity: “ *Thus, our results are in line with these studies, showing an effect of sleep disturbances on SMM in a Latin American population*”.  

***Comment:* In Table 2, it is not clear for which measurements the p values ​​are. Table 2 is inadequate and incomplete in showing the variables associated with sleep disorders.**

**Response: **Table 2 follows the same structure presented in Table 1, but in this case, we are comparing two variables (at the top of the table): sleep difficulties and awakening at nights. As a result, we have to p-value columns, the first one for each comparison done with sleep difficulties, and the second one for the comparisons done with the other variable. Moreover, the style of the journal is complicated as the extension of Table 2 is a little more than 2 pages. According to the reviewer, Table 2 can be incomplete as we do not show all the confounders, but not all the variables (as requested by the reviewer) are available as they were not collected as part of the original intervention study (explained in a previous comment).

# Reviewer report: Reviewer response for version 3

**DOI:** 10.21956/wellcomeopenres.25820.r113032

**Published:** 2025-01-23

**Authors:** Helton de Sá Souza

**Affiliations:** 1 Universidade Federal de Vicosa, Viçosa, State of Minas Gerais, Brazil

This is a study that aimed to assess the association between sleep duration and disturbances (sleep difficulties and awakening at nights) and changes of SMM over time in a resource-constrained Peruvian population. However, the authors do not present sample calculations;

 The authors do not provide details on how the volunteers were accessed, whether the sample is random or convenience.

 It is not clear whether the authors used validated questionnaires or whether they created new instruments, even if based on others, without prior validation. This needs to be clear.

 In a population study focused on sleep parameters, it's vital to understand that merely tracking the quantity of sleep does not provide the necessary insights for informed clinical interventions or meaningful public policy development. To create impactful solutions, must identify and address the most prevalent sleep disorders affecting the population. Apparently, the instruments used for data collection weaken the broader understanding of sleep in the study population.

 The study was conducted in South America, home to one of the world's leading centers for sleep research, which is responsible for significant epidemiological studies on sleep. However, the study did not reference the works generated by EPISONO. I believe that at least three studies developed by the Brazilian group should be considered.

 SANTOS-SILVA et al, 2010 - doi.org/10.1016/j.sleep.2009.12.011

 PIOVEZAN et al, 2019 - doi 10.1002/jcsm.1244,

 DE SÁ SOUZA et al 2022 - doi.org/10.3390/ijerph192316322

Is the work clearly and accurately presented and does it cite the current literature?

Partly

If applicable, is the statistical analysis and its interpretation appropriate?

I cannot comment. A qualified statistician is required.

Are all the source data underlying the results available to ensure full reproducibility?

Yes

Is the study design appropriate and is the work technically sound?

Partly

Are the conclusions drawn adequately supported by the results?

Partly

Are sufficient details of methods and analysis provided to allow replication by others?

No

Reviewer Expertise:

Sleep Science; Exercise, Endurance training

I confirm that I have read this submission and believe that I have an appropriate level of expertise to confirm that it is of an acceptable scientific standard, however I have significant reservations, as outlined above.

## References

[1] : Increasing trends of sleep complaints in the city of Sao Paulo, Brazil. *Sleep Med* .2010;11(6) : 10.1016/j.sleep.2009.12.011 520-4 10.1016/j.sleep.2009.12.011 20494615

[2] : Associations between sleep conditions and body composition states: results of the EPISONO study. *J Cachexia Sarcopenia Muscle* .2019;10(5) : 10.1002/jcsm.12445 962-973 10.1002/jcsm.12445 31125517 PMC6818458

[3] : Resistance Training Improves Sleep and Anti-Inflammatory Parameters in Sarcopenic Older Adults: A Randomized Controlled Trial. *Int J Environ Res Public Health* .2022;19(23) : 10.3390/ijerph192316322 10.3390/ijerph192316322 36498393 PMC9736460

# Response

**Published:** 2025-01-25

**Authors:** Antonio Bernabe-Ortiz

**Affiliations:** Universidad Cientifica del Sur, Peru

This is a study that aimed to assess the association between sleep duration and disturbances (sleep difficulties and awakening at nights) and changes of SMM over time in a resource-constrained Peruvian population. **The authors do not present sample calculations.** **Response: **We have now included the calculation of the power of the study.  In the Methods section, now it reads: “ *With at least 360 participants in each sleep group (i.e., those with and without sleep difficulties), we had a power over 90% to detect a difference in SMM of 0.5 Kg (i.e., 18.3 kg among those without sleep difficulties vs. 17.7 Kg among those with sleep difficulties) at the end of follow-up, assuming a SD of 4.1 and a correlation between follow-up measurements of 0.7. Since our database had 2310 participants at baseline, we ensure enough power to find differences in our analysis.*”  

**The authors do not provide details on how the volunteers were accessed, whether the sample is random or convenience.**

***Response:*** Mid-sized villages with 350–700 individuals (~130–250 households) were initially selected for the study. Of the 20 mid-size villages available with these characteristics, six were randomly selected. After that, a census was conducted to enroll all the participants aged 18+ years, full-time residents in the area, capable of understanding procedures and providing informed consent. Now it reads: *“Mid-sized villages with 350–700 individuals (~130–250 households) were initially selected for the study. Of the 20 mid-size villages available with these characteristics, six were randomly selected. After that, a census strategy was applied to invite and enroll potential participants.”  *  

**It is not clear whether the authors used validated questionnaires or whether they created new instruments, even if based on others, without prior validation. This needs to be clear.**

***Response:*** We have used validated tools as explained in the variable’s description. However, we have added details about the questionnaires in the Procedures section. Now it reads: “ *Questionnaires were created using previously validated tools such as the WHO STEPwise approach to NCD risk factor surveillance (STEPS) for lifestyles, the Sleep Heart Health Study, the Obstructive Sleep Apnea questionnaire, the International Physical Activity Questionnaire, and the Patient Health Questionnaire-9. Follow-up evaluations were carried out every five months using the same protocol and tools.*”  

***In a population study focused on sleep parameters, it's vital to understand that merely tracking the quantity of sleep does not provide the necessary insights for informed clinical interventions or meaningful public policy development. To create impactful solutions, must identify and address the most prevalent sleep disorders affecting the population. Apparently, the instruments used for data collection weaken the broader understanding of sleep in the study population.***

***Response:*** We agree with the reviewer, but as we explained before, this is a secondary data analysis using information of an intervention focused on hypertension.  Thus, the study was not specifically developed to assess the effect of sleep patterns on skeletal muscle mass. This has been pointed out in the limitations section: “ *First, the data used in the present manuscript comes from a population-based intervention using a salt substitute…*”  

**The study was conducted in South America, home to one of the world's leading centers for sleep research, which is responsible for significant epidemiological studies on sleep. However, the study did not reference the works generated by EPISONO. I believe that at least three studies developed by the Brazilian group should be considered:** SANTOS-SILVA et al, 2010 - doi.org/10.1016/j.sleep.2009.12.011 PIOVEZAN et al, 2019 - doi 10.1002/jcsm.1244, DE SÁ SOUZA et al 2022 - doi.org/10.3390/ijerph192316322

***Response:*** We would like to thank the reviewer for these references. We have decided to include two of them in our manuscript. Thus, in the Introduction, we have added: “ *A study in Brazil evaluating the trends of sleep complaints at the population level in Sao Paulo, reported a progressive increase of sleep complaints throughout three consecutive decades*”. Whereas in the Discussion, we have added: “ *For example, a trial conducted in Sao Paulo and using resistance exercise training for 12 weeks improved simultaneously sleep parameters and muscle performance*”.

# Reviewer report: Reviewer response for version 3

**DOI:** 10.21956/wellcomeopenres.25820.r113852

**Published:** 2024-12-16

**Authors:** Nurel Ertürk

**Affiliations:** 1 University of Health Sciences, Trabzon, Turkey

The literature and physiological background of the study are lacking. We can see this in many ways:

**1- **Many factors such as hormones that will affect women's muscle mass, menopause, breastfeeding, and hysterectomy surgeries affect muscle mass. These are confounding factors when researching muscle mass. These have not been looked at.

**2- **In both the Summary and Results sections, only the number of female patients is written, and the number of male patients should be added. In the measurements made, sleep disturbance and sleep duration were evaluated completely subjectively and according to patient responses. Sleep duration has many sub parameters. Such as the hours spent in bed, the hours spent in bed and getting out of bed, whether the person walked around the house at night and stayed awake during the sleep time... It is an unreliable measurement to evaluate sleep only with subjective measurement and even with two questions. The biggest shortcoming of this study is that sleep measurements are very inadequate and unreliable.

 "Thus, our results expand literature by showing a longitudinal effect of sleep disturbances on SMM in a Latin American population." You say that there is no relationship in your results, and you also did not examine many confounding factors. Based on what conclusion do you say this? In Table 2, it is not clear for which measurements the p values ​​are. Table 2 is inadequate and incomplete in showing the variables associated with sleep disorders.

Is the work clearly and accurately presented and does it cite the current literature?

No

If applicable, is the statistical analysis and its interpretation appropriate?

Partly

Are all the source data underlying the results available to ensure full reproducibility?

No

Is the study design appropriate and is the work technically sound?

No

Are the conclusions drawn adequately supported by the results?

No

Are sufficient details of methods and analysis provided to allow replication by others?

Yes

Reviewer Expertise:

Sleeo health, obstructive sleep apnea, physical activity

I confirm that I have read this submission and believe that I have an appropriate level of expertise to state that I do not consider it to be of an acceptable scientific standard, for reasons outlined above.

# Reviewer report: Reviewer response for version 3

**DOI:** 10.21956/wellcomeopenres.25820.r111649

**Published:** 2024-11-18

**Authors:** Laura Žlibinaite

**Affiliations:** 1 Kauno Kolegija Higher Education Institution, Kaunas, Lithuania

Thank you for the revised version and corresponding the comments. I have no further comments to make.

Is the work clearly and accurately presented and does it cite the current literature?

Partly

If applicable, is the statistical analysis and its interpretation appropriate?

I cannot comment. A qualified statistician is required.

Are all the source data underlying the results available to ensure full reproducibility?

Yes

Is the study design appropriate and is the work technically sound?

Partly

Are the conclusions drawn adequately supported by the results?

Partly

Are sufficient details of methods and analysis provided to allow replication by others?

Partly

Reviewer Expertise:

Physiotherapy, exercise physiology

I confirm that I have read this submission and believe that I have an appropriate level of expertise to confirm that it is of an acceptable scientific standard.

# Reviewer report: Reviewer response for version 1

**DOI:** 10.21956/wellcomeopenres.25413.r105133

**Published:** 2024-11-01

**Authors:** Laura Žlibinaite

**Affiliations:** 1 Kauno Kolegija Higher Education Institution, Kaunas, Lithuania

The article "Sleep duration, sleep disturbances and skeletal muscle mass change over time: A population-based longitudinal analysis in Peru“ written by Agurto-García and colleagues provides a longitudinal observational data of the relationship between sleep patterns and skeletal muscle mass (SMM) in Peruvian population. The findings underline the impact of sleep duration and disturbances on SMM loss over time. Even though the study represents a significant data, some parts of the manuscript require changes to improve the overall soundness and quality.

1. **Introduction:**
  1. The background information regarding study concerns (skeletal muscles, sarcopenia) needs deeper literature analysis. Even the first sentence seems misleading, because the main function of skeletal muscle is not only structural, but mostly movement.
  2. Sentence “This latter report also stated the scarce data assessing such association in resource-constrained settings, as well as the lack of longitudinal evaluations” is barely comprehensible.
  3. The generalization of the findings from studies of sleep disturbances is almost impossible, because sarcopenia is age related condition. The authors should emphasize what is their hypothesis to analyze data of young adults regarding loss of muscle mass.
  4. There is lack of background information about the factors influencing sleep quality. How hypertension is related with current study?
  5. The same applies to the analyzing what has been already found about risk factors for sarcopenia.
2. **Methods:**
  1. The study uses two methods, bioimpedance and the Lee’s formula, to assess skeletal muscle mass (SMM). However, the authors do not sufficiently explain why there are discrepancies between these methods, especially among individuals who are long sleepers. As the researchers should be available of, the biompedance analysis has a strict protocol of the assessment procedure. Where is this explained? A more detailed analysis of why bioimpedance indicates a notable reduction in SMM while the formula does not would add clarity and strengthen the findings.
  2. What is meant by the paragraph subtitle “ *exposures*”?
  3. Why the education level was expressed by years?
  4. The explanation of who did the weight and height assessment should be provided.
3. **Results:**
  1. Table 1, Table 2 and Table 3 represents baseline data. Given the information that evaluations were conducted at the baseline and 5 subsequent times, the reader misses a part of information.
  2. The results from Table 3 are contradictory, cite: “SMM was higher among those smoking daily, those with high alcohol consumption, those with moderate/high physical activity, and obese subjects” and requires special explanation in the discussion. Does it lead to the unhealthy lifestyle?
  3. Data changes during time should be analyzed with the age taken into consideration. It is not appropriate to generalize data from this study and only separate participants by sleep time and sleep disturbances. It is well-established that usually skeletal muscle mass declines with age. So results could be absolutely controversial when analyzed by age group.
4. **Discussion:**
  1. Please clarify, what was the idea to comment on DEXA analysis used in the previous studies, if this study does not use the same body composition measurement?
  2. Authors write: “Regarding cross-sectional studies, in a sample of 488 Taiwanese adults, a report showed a significant association between sleep duration and the prevalence of sarcopenia”. In my opinion, would be more beneficial to comment on what were those associations.
  3. To my understanding, this study does expand the literature, but not explain the potential reasons and correlations. Because the results are contrary to what was explained in the previous literature, it would be beneficial to discuss what determined these differences.
5. Public health relevance should be referencing to recommendations regarding sleep hygiene and do not stepping into topics outside the scopus of this study (i.e. dietary strategies).
6. **Strengths and limitations:**
  1. I would consider wide age group more life limitation, but not strength. It would improve the quality of the study if authors would do age-related analysis.
  2. Another limitation is that covariates like dietary intake (especially protein consumption), physical activity habits, and psycho-emotional state were not considered, which may significantly impact SMM outcomes.

Is the work clearly and accurately presented and does it cite the current literature?

Partly

If applicable, is the statistical analysis and its interpretation appropriate?

I cannot comment. A qualified statistician is required.

Are all the source data underlying the results available to ensure full reproducibility?

Yes

Is the study design appropriate and is the work technically sound?

Partly

Are the conclusions drawn adequately supported by the results?

Partly

Are sufficient details of methods and analysis provided to allow replication by others?

Partly

Reviewer Expertise:

Rehabilitation

I confirm that I have read this submission and believe that I have an appropriate level of expertise to confirm that it is of an acceptable scientific standard, however I have significant reservations, as outlined above.

# Response

**Published:** 2024-11-11

**Authors:** Antonio Bernabe-Ortiz

**Affiliations:** Universidad Cientifica del Sur, Peru

**Introduction:** 1. The background information regarding study concerns (skeletal muscles, sarcopenia) needs deeper literature analysis. Even the first sentence seems misleading, because the main function of skeletal muscle is not only structural, but mostly movement. ***Response:*** We have rewritten this section to include the function of skeletal muscle. Now it reads: “ *The skeletal muscle has different functions, including produce movement, sustain body posture and position, maintain body temperature, and stabilize joints, but it also plays an important role in human metabolism as it is the main tissue responsible for insulin-mediated glucose storage, in addition to consuming a significant amount of oxygen*”.  

 2. Sentence “This latter report also stated the scarce data assessing such association in resource-constrained settings, as well as the lack of longitudinal evaluations” is barely comprehensible.

***Response:*** This part has been rewritten for clarity: “ *This review highlighted the lack of longitudinal information assessing the association between sleep patterns and skeletal muscle mass in resource-constrained settings*”.  

 3. The generalization of the findings from studies of sleep disturbances is almost impossible, because sarcopenia is age related condition. The authors should emphasize what their hypothesis is to analyze data of young adults regarding loss of muscle mass.

***Response:*** We have added a sentence regarding this point: “ *Subjects typically start to lose muscle mass and strength in their 30 or 40 years, but the rate of decline increases during elderly. Thus, most of the studies have been focused on sarcopenia on elderly individuals, instead of reduction of muscle mass in the general adult population*”.  

 4. There is lack of background information about the factors influencing sleep quality. How is hypertension related to current study?

***Response:*** According to literature many chronic conditions are associated with sleep quality e.g., hypertension, type 2 diabetes, chronic kidney disease, etc.). Even multimorbidity (i.e., multiple long-term conditions) has been associated with poor sleep quality. Thus, we disagree with the reviewer regarding this point as we do not see a reason to dedicate part of the introduction to hypertension and not the other conditions.  

 5. The same applies to analyzing what has been already found about risk factors for sarcopenia.

***Response***: We have rewritten this section to add risk factors for sarcopenia: “ *Ageing has a direct effect on the loss of skeletal muscle mass (SMM) in humans; however, the reduction of skeletal muscle mass is also related to a variety of factors including malnutrition, low physical activity, and sleep patterns, including sleep duration and quality*”.  

**Methods:** 1. The study uses two methods, bioimpedance and the Lee’s formula, to assess skeletal muscle mass (SMM). However, the authors do not sufficiently explain why there are discrepancies between these methods, especially among individuals who are long sleepers. As the researchers should be available of, bioimpedance analysis has a strict protocol of the assessment procedure. Where is this explained? A more detailed analysis of why bioimpedance indicates a notable reduction in SMM while the formula does not would add clarity and strengthen the findings.

***Response:*** A standard of procedures (SOP) was created in Spanish for bioimpedance analysis, based on the manufacturer’s manual of the TANITA TBF-300A. In addition, training of fieldworkers in procedures was carried out before any fieldwork activities. We have included that in the Methods section: “ *A standard of procedures (SOP) was created in Spanish based on the manufacturer’s manual of the TANITA TBF-300A”, and “A training course for fieldworkers focused on informed consent, questionnaires application, and procedures was carried out before any fieldwork activities*”. The inclusion of potential reasons for discrepancies between the methods used was added after reviewer 1 requested that change, and we are showing this now as the reviewer evaluated a previous version of the paper. We included that in the last paragraph of the Comparison with previous studies section: “ *Finally, although the correlation between results by bioimpedance and the Lee’s formula was high, estimations of SMM and its changes over time were divergent in some cases. Some potential reasons for this are that the algorithm used to estimate SMM when applying bioimpedance may not be applicable to Latin America populations [a], and for instance increase the risk of measurement bias. That is not the case when using the Lee’s formula, previously validated for Latin American population. Besides, the bioimpedance device (TANITA TBF-300A) used for this study was not the same as the original one [b], implying measurement bias as different devices may use different estimation algorithms*”. ***References:*** [a] Fernandez Veitez JA, Aguilera RR: [Muscle mass estimation by different anthropometric equations in high performance weightlifters]. Archivos de Medicina del Deporte. 2001;XVIII(86):585–591. [b] Rangel Peniche DB, Raya Giorguli G, Alemán-Mateo H: Accuracy of a predictive bioelectrical impedance analysis equation for estimating appendicular skeletal muscle mass in a non-Caucasian sample of older people. Arch Gerontol Geriatr. 2015;61(1):39–43. 25857600 10.1016/j.archger.2015.03.007  

 2. What is meant by the paragraph subtitle “exposures”?

***Response:*** This is a manuscript reporting a relationship between sleep patterns and changes of skeletal muscle mass. Therefore, there is a dependent (or outcome) and an independent (or exposure) variable. In this case, the outcome is skeletal muscle mass evaluated using two different techniques, and different exposures (sleep duration and sleep disturbances). We have expanded the sentence to clarify this point: “ *Two were the independent (i.e., exposure) variables used in the analysis: sleep duration and sleep disturbances (sleep difficulties and awakening at nights)*”.  

 3. Why was the education level expressed by years?

***Response:*** The definition used for education level is based on the organization of the education system in Peru (primary, secondary and superior). Thus, primary education represents less than 7 years of education, secondary education is between 7 and 11 years of education, and superior education is that of 12 or more years of education. We have included that information for clarity. Now it reads: “ *… education level (primary [<7 years], secondary [7–11 years], and superior [12+ years]), …*”  

 4. The explanation of who did the weight and height assessment should be provided. ***Response***: We have added that information in the Procedures and data collection section: “ *Height was measured using a stadiometer, with the participant in standing position and without shoes. The Frankfurt’s plane was used to guarantee appropriate position of the head. On the other hand, weight was obtained from the TANITA TBF-300A Total Body composition analyzer, in standing position, and without shoes*”.  

**Results:** 1. Table 1, Table 2 and Table 3 represent baseline data. Given the information that evaluations were conducted at the baseline and 5 subsequent times, the reader misses a part of information.

***Response:*** We request the reviewer to suggest what analysis is needed. Usually, the report of the outcome and exposure are needed, and in the tables the characterization of other variables according to the variables of interest is mandatory. Showing the crude variation of the SMM over time is not usual.  

 2. The results from Table 3 are contradictory, cite: “SMM was higher among those smoking daily, those with high alcohol consumption, those with moderate/high physical activity, and obese subjects” and requires special explanation in the discussion. Does it lead to an unhealthy lifestyle?

***Response:*** We have reviewed our analyses, and they are correct. These apparently contradictory results are potentially due to: (a) the analysis of cross-sectional variables in Table 3, so, reverse causality can be an issue here; and (b) they are only crude (unadjusted models) and for instance these are not the final results. Because there is no interest in these variables but only as potential confounders, results may be interpreted cautiously.  

 3. Data changes during time should be analyzed with the age taken into consideration. It is not appropriate to generalize data from this study and only separate participants by sleep time and sleep disturbances. It is well-established that usually skeletal muscle mass declines with age. So, results could be absolutely controversial when analyzed by age group.

***Response:*** Age is part of the models; it has been included as a potential confounder. What the reviewer suggests is that age is a potential effect modifier of the association, i.e., the association is stronger among elderly compared to young adults. This is not part of the proposed manuscript as usually a greater number of records is needed to ensure appropriate power to detect effect modification.  

**Discussion:** 1. Please clarify, what was the idea to comment on DEXA analysis used in the previous studies, if this study does not use the same body composition measurement? ***Response:*** DEXA is the gold standard in the case of body composition measurement, and results are part of the systematic review being discussed in that section. The point to include a comment of DEXA is because: “ *DEXA and bioimpedance may be difficult to utilize in resource-constrained settings, and DEXA might overestimate sarcopenia*” as it has been included in that section.  

 2. Authors write: “Regarding cross-sectional studies, in a sample of 488 Taiwanese adults, a report showed a significant association between sleep duration and the prevalence of sarcopenia”. In my opinion, it would be more beneficial to comment on what those associations were.

***Response:*** We have added details about this manuscript: “ *Regarding cross-sectional studies, in a sample of 488 Taiwanese elderly adults, a report showed a significant U-shaped association between sleep duration and the prevalence of sarcopenia. Thus, compared to adults with 6-8 h of sleep, elderly subjects with <6 h of sleep had a nearly 3-fold increased probability of sarcopenia, whereas elderly with ≥8 h of sleep had a nearly 2-fold increased risk of sarcopenia*”.  

 3. To my understanding, this study does expand the literature but does not explain the potential reasons and correlations. Because the results are contrary to what was explained in the previous literature, it would be beneficial to discuss what determined these differences. ***Response:*** We have added different information in the Discussion section as requested by the two reviewers.  

 4. Public health relevance should be referencing to recommendations regarding sleep hygiene and do not stepping into topics outside the scope of this study (i.e. dietary strategies). ***Response:*** We have rewritten this section. Diet is also a potential way to deal with SMM reduction. Now it reads: “ *Thus, an appropriate strength-training routine could be used, involving a well-qualified trainer to help set up a detailed sequence and supervise workouts. Diet improvement is also needed, for example, adequate protein and/or amino-acid intake plus exercise can enhance the benefits. Vitamin D, omega-3s, and dairy may positively impact muscle mass and strength*”,  

**Strengths and limitations:** 1. I would consider wide age group more like limitation, but not strength. It would improve the quality of the study if authors would do age-related analysis. ***Response:*** As explained above, we believe evaluating a wide age group is a strength as muscle mass reduction starts as early as 30 years old. We decided not to change this section.  

 2. Another limitation is that covariates like dietary intake (especially protein consumption), physical activity habits, and psycho-emotional state were not considered, which may significantly impact SMM outcomes.

***Response:*** This was also highlighted by the other reviewer. We have modified the manuscript and different sections to include psychosocial-emotional state as the presence of depressive symptoms (as a confounder). We have also added a sentence in the limitations section to include the other covariates not available in the database: “ *Finally, some confounders of the association of interest may have not been included as they were not available, such as the presence of anxiety symptoms, diet patterns, especially meat consumption, protein consumption, physical activity intensity, among others*”.

# Reviewer report: Reviewer response for version 1

**DOI:** 10.21956/wellcomeopenres.25413.r104052

**Published:** 2024-10-15

**Authors:** Yuquan Chen

**Affiliations:** 1 School of Public Health and Preventive Medicine, Monash University, Melbourne, Victoria, Australia

The article, titled *"Sleep duration, sleep disturbances and skeletal muscle mass change over time: A population-based longitudinal analysis in Peru"*, provides an insightful examination of the relationship between sleep patterns and skeletal muscle mass (SMM) in a resource-limited setting. The authors utilized data from a well-designed population-based longitudinal study to explore these associations. The findings highlight the impact of sleep disturbances on muscle mass decline over time. While the study contributes valuable data, several areas require substantial improvement to strengthen its overall quality and scientific rigor.

**1. Measurement Inconsistency:** The study relies on two methods (bioimpedance and a validated formula) for assessing SMM. However, the authors do not adequately address why discrepancies exist between these methods, particularly in long sleepers. Providing an analysis of why bioimpedance shows a significant reduction while the formula does not would be beneficial.

**2. Limited Control of Confounding Variables:** While the authors adjusted their models for several covariates, important factors such as dietary intake (e.g., protein consumption), physical activity intensity, and psychological factors like depression or anxiety were not considered. These variables could significantly influence SMM and should be incorporated into the models to provide more accurate results.

**3. Use of Self-reported Sleep Data:** The reliance on self-reported sleep duration and disturbances raises concerns about the validity of the findings. Self-reported measures are prone to recall bias. Incorporating objective measures, such as actigraphy, would enhance the reliability of sleep assessments and offer a more robust evaluation.

**4. Insufficient Detail on Attrition and Its Impact:** The study mentions a 12.1% attrition rate but does not elaborate on how this may have influenced the results. The authors should conduct and report sensitivity analyses to determine whether the dropouts significantly impacted the findings, especially considering the socioeconomic and demographic characteristics of the participants who left the study.

**5. Lack of Specificity in the Description of the Population:** The paper provides general information about the sample, but more details on socioeconomic diversity, physical activity levels, and nutrition would enrich the context. Given the regional and socioeconomic variability in Peru, these aspects are crucial for understanding the generalizability of the findings.

**6. Potential Bias from the Initial Intervention: **The study uses data from an intervention aimed at reducing salt intake. Although a post-hoc analysis is mentioned, there is insufficient detail on how the salt intervention may have interacted with sleep patterns or muscle mass. A thorough analysis to differentiate the effects of the intervention from the variables studied is necessary.

**7. Inadequate Exploration of Gender Differences:** The article indicates that SMM and sleep disturbances differ between men and women, yet the analysis does not explore these differences further. Given the known disparities in sleep patterns and muscle composition between genders, the authors should provide a stratified analysis to investigate if these associations hold true across both sexes.

**8. Unclear Public Health Implications:** The discussion section makes vague references to the importance of sleep hygiene and public health interventions. It would be more effective if the authors linked their findings directly to specific recommendations for improving muscle health through sleep management in resource-limited settings, outlining feasible interventions or policies.

 These issues collectively suggest the need for a major revision. Addressing them would not only enhance the rigor of the study but also provide more clarity and applicability to the findings.

Is the work clearly and accurately presented and does it cite the current literature?

Yes

If applicable, is the statistical analysis and its interpretation appropriate?

Yes

Are all the source data underlying the results available to ensure full reproducibility?

Yes

Is the study design appropriate and is the work technically sound?

Partly

Are the conclusions drawn adequately supported by the results?

Partly

Are sufficient details of methods and analysis provided to allow replication by others?

Partly

Reviewer Expertise:

physical activity

I confirm that I have read this submission and believe that I have an appropriate level of expertise to confirm that it is of an acceptable scientific standard, however I have significant reservations, as outlined above.

# Response

**Published:** 2024-10-28

**Authors:** Antonio Bernabe-Ortiz

**Affiliations:** Universidad Cientifica del Sur, Peru

**1. Measurement Inconsistency:** The study relies on two methods (bioimpedance and a validated formula) for assessing SMM. However, the authors do not adequately address why discrepancies exist between these methods, particularly in long sleepers. Providing an analysis of why bioimpedance shows a significant reduction while the formula does not would be beneficial.

***Response:*** We have added that discussion in the final paragraph of comparisons with previous studies section: “ *Finally, although the correlation between results by bioimpedance and the Lee’s formula was high, estimations of SMM and its changes over time were divergent in some cases. Some potential reasons for this are that the algorithm used to estimate SMM when applying bioimpedance may not be applicable to Latin America populations, and for instance increase the risk of measurement bias. That is not the case when using the Lee’s formula, previously validated for Latin American population. Besides, the bioimpedance device (TANITA TBF-300A) used for this study was not the same as the original one (a), implying measurement bias as different devices may use different estimation algorithms*”.

***References:*** a. Rangel Peniche DB et al. Accuracy of a predictive bioelectrical impedance analysis equation for estimating appendicular skeletal muscle mass in a non-Caucasian sample of older people. *Arch Gerontol Geriatr.* 2015;61(1):39–43.

**2. Limited Control of Confounding Variables:** While the authors adjusted their models for several covariates, important factors such as dietary intake (e.g., protein consumption), physical activity intensity, and psychological factors like depression or anxiety were not considered. These variables could significantly influence SMM and should be incorporated into the models to provide more accurate results.

***Response:*** Unfortunately, variables such as protein consumption or intensity of physical activity were not collected in the original study, and for instance, were not available for analysis. We have added this topic to the limitations section. However, depressive symptoms were collected using the PHQ-9 tool, and therefore, were included in the analyses and the other sections of the manuscript accordingly. Changes in estimates were only marginal, and did not affect the results or their interpretation.

**3. Use of Self-reported Sleep Data:** The reliance on self-reported sleep duration and disturbances raises concerns about the validity of the findings. Self-reported measures are prone to recall bias. Incorporating objective measures, such as actigraphy, would enhance the reliability of sleep assessments and offer a more robust evaluation.

***Response:*** We agree with the reviewer regarding this point. However, objective measures were not available to measure sleep variables. We have added such concern in the limitations section as follows: “ *Fourth, sleep duration and disturbances were measured using a self-reported technique, a method that can be less accurate than actigraphy, and for instance, recall and measurement biases may arise*”.

**4. Insufficient Detail on Attrition and Its Impact:** The study mentions a 12.1% attrition rate but does not elaborate on how this may have influenced the results. The authors should conduct and report sensitivity analyses to determine whether the dropouts significantly impacted the findings, especially considering the socioeconomic and demographic characteristics of the participants who left the study.

***Response:*** We agree with the reviewer regarding this point as attrition rates may affect findings and generalizability. We have conducted an analysis comparing those who were kept in the study and those who lost to follow-up. We have added the results as text in the appropriate section: “ *Those who lost to follow-up were younger, had higher education, had low levels of physical activity, had BMI < 30 kg/m2, and had more depressive symptoms at baseline than those who were kept in the study*”. In addition, a comment has been added to the Limitations section regarding this issue: “ *In addition, attrition rates may have an impact on our results, especially because those lost to follow-up were different from those who were kept in the study”*.

**5. Lack of Specificity in the Description of the Population:** The paper provides general information about the sample, but more details on socioeconomic diversity, physical activity levels, and nutrition would enrich the context. Given the regional and socioeconomic variability in Peru, these aspects are crucial for understanding the generalizability of the findings. ***Response:*** We have added information regarding the context and setting in the Methods section: “ *The study was conducted in Tumbes, a coastal region in northern Peru, in the border with Ecuador, with 225 thousand inhabitants, and where approximately 90% of the population is urban. Additionally, nearly 20% and 1% of the population are considered poor and extremely poor, respectively, and 15% do not have basic sanitation services.” *We have, in addition, added a reference regarding this point.

**6. Potential Bias from the Initial Intervention: **The study uses data from an intervention aimed at reducing salt intake. Although a post-hoc analysis is mentioned, there is insufficient detail on how the salt intervention may have interacted with sleep patterns or muscle mass. A thorough analysis to differentiate the effects of the intervention from the variables studied is necessary.

***Response:*** We have now included a variable for salt substitution implementation over time in our model as the original study used a stepped wedge design. Despite this, results are very similar to the originally showed. In addition, we have added a brief discussion regarding this point: “ *However, we adjusted our models for the intervention and the moment at which the salt substitute was implemented as the original study had a stepped wedge design. A previous study reported that salt substitutes were believed to improve sleeping disorders and musculoskeletal health but was considered an exaggeration of the benefits of the substitute (a). A different study reported that short-duration sleepers consumed insufficient amounts of vegetables but preferred salty food (b). Literature suggests that mechanisms playing a role in the association between sleep duration and dietary intake are likely to be multifactorial and include differences in the appetite-related hormones (c)*”.

***References:*** a. Liu Y et al. Factors associated with the use of salt substitute in rural China. JAMA Netw Open 2021;4(12):e2137745. b. Shi Z et al. Dietary fat and sleep duration in Chines men and women. Int J Obes (Lond) 2008;32(12):1835-40. c. Dashti HS et al. Short sleep duration and dietary intake: epidemiologic evidence, mechanisms, and health implications. Adv Nutr 2015;6(6):648-59.

**7. Inadequate Exploration of Gender Differences:** The article indicates that SMM and sleep disturbances differ between men and women, yet the analysis does not explore these differences further. Given the known disparities in sleep patterns and muscle composition between genders, the authors should provide a stratified analysis to investigate if these associations hold true across both sexes.

***Response:*** Whereas it is true that SMM and sleep disturbances are not similar among men and women, the evaluation of sex as a possible effect modifier of the association of interest is not an aim of the manuscript. We have, however, included a couple of sentences regarding this point. For example, in the Statistical analysis section we added: “ *Finally, as SMM and sleep duration and disturbances differ between men and women, we evaluated if sex was an effect modifier (i.e., interaction) of the associations of interest*”. In addition, we have modified the results and discussions section accordingly.

**8. Unclear Public Health Implications:** The discussion section makes vague references to the importance of sleep hygiene and public health interventions. It would be more effective if the authors linked their findings directly to specific recommendations for improving muscle health through sleep management in resource-limited settings, outlining feasible interventions or policies.

***Response:*** This is an observational study, and for instance, results may be interpreted cautiously. Nevertheless, we have improved our section: “ *Therefore, our findings suggest the need to maintain appropriate sleep patterns to preserve and avoid loss of muscle mass. Appropriate prevention strategies are also mandatory as usually sleep quality and duration tend to reduce during ageing, whereas time awake increases after sleep onset. Thus, an appropriate strength-training routine could be used, involving a well-qualified trainer to help set up a detailed sequence and supervise workouts. Diet improvement is also needed, for example, adequate protein and/or amino-acid intake plus exercise can enhance the benefits. Vitamin D, omega-3s, and dairy may positively impact muscle mass and strength*.”
